# Failure to Rescue After Resection of Perhilar Cholangiocarcinoma in an International Multicenter Cohort

**DOI:** 10.1245/s10434-024-16293-7

**Published:** 2024-10-15

**Authors:** Pim B. Olthof, Stefan A. W. Bouwense, Jan Bednarsch, Maxime Dewulf, Geert Kazemier, Shishir Maithel, William R. Jarnagin, Luca Aldrighetti, Keith J. Roberts, Roberto I. Troisi, Massimo M. Malago, Hauke Lang, Ruslan Alikhanov, Andrea Ruzzenente, Hassan Malik, Ramón Charco, Ernesto Sparrelid, Johann Pratschke, Matteo Cescon, Silvio Nadalin, Jeroen Hagendoorn, Erik Schadde, Frederik J. H. Hoogwater, Andreas A. Schnitzbauer, Baki Topal, Peter Lodge, Steven W. M. Olde Damink, Ulf P. Neumann, Bas Groot Koerkamp, on behalf of the Perihilar Cholangiocarcinoma Collaboration Group, F. Bartlett Dm Bartsch, W. O. Bechstein, J. Bednarsch, C. Benzing, M. T. de Boer, S. Buettner, I. Capobianco, M. I. D’Angelica, P. de Reuver, E. de Savornin Lohman, C. Dopazo, M. Efanov, J. I. Erdmann, L. C. Franken, J. Geers, M. C. Giglio, S. Gilg, C. Gomez-Gavara, A. Guglielmi, T. M. van Gulik, A. Hakeem, J. Heil, H. Jansson, T. P. Kingham, S. K Maithel, R. Margies, R. Marino, Q. I. Molenaar, T. A. Nguyen, L. E. Nooijen, C. L. M. Nota, E. Poletto, R. J. Porte, R. Prasad, L. M. Quinn, F. Ratti, M. Ravaioli, J. Rolinger, M. Schmelzle, M. Serenari, A. Sultana, R. Sutcliff, H. Topal, S. van Laarhoven, B. M. Zonderhuis

**Affiliations:** 1 Department of Surgery, Erasmus Medical Center, Rotterdam, The Netherlands; 2 Department of Surgery, Amsterdam UMC, Amsterdam, The Netherlands; 3 Department of Surgery, University Medical Center Groningen, Groningen, The Netherlands; 4 Department of Surgery, Maastricht University Medical Center+, Maastricht, The Netherlands; 5 Department of General, Visceral and Transplantation Surgery, Universitats Klinikum Essen, Essen, Germany; 6 Division of Surgical Oncology, Winship Cancer Institute, Emory University, Atlanta, GA USA; 7 Hepatopancreatobiliary Service, Department of Surgery, Memorial Sloan Kettering Cancer Center, New York, NY USA; 8 Hepatobiliary Surgery Division, IRCCS San Raffaele Scientific Institute and Vita-Salute San Raffaele Univeristy, Milan, Italy; 9 Department of Surgery, University Hospital Birmingham, Birmingham, UK; 10 Division of HPB, Minimally Invasive and Robotic Surgery, Transplantation Service, Federico II University Hospital Naples, Naples, Italy; 11 Department of HPB- and Liver Transplantation Surgery, Royal Free Hospitals, University College London, London, UK; 12 Department of General, Visceral and Transplantation Surgery, University Medical Center, Mainz, Germany; 13 Department of Liver and Pancreatic Surgery, Department of Transplantation, Moscow Clinical Scientific Centre, Moscow, Russia; 14 Department of Surgery, Division of General Surgery, Unit of Hepato-Pancreato-Biliary Surgery, University of Verona Medical School, Verona, Italy; 15 Liver Surgery Unit, Aintree University Hospital, Liverpool, UK; 16 Department of HBP Surgery and Transplantation, Hospital Universitario Vall d’Hebron, Universidad Autónoma de Barcelona, Barcelona, Spain; 17 Division of Surgery and Oncology, Department for Clinical Science, Intervention and Technology, Karolinska Institutet, Karolinska University Hospital, Stockholm, Sweden; 18 Department of Surgery, Campus Charité Mitte and Campus Virchow-Klinikum, Charité-Universitätsmedizin Berlin, Berlin, Germany; 19 General Surgery and Transplant Unit, IRCCS- Azienda Ospedaliero-Universitaria di Bologna, Bologna, Italy; 20 Department of General and Transplant Surgery, University Hospital Tübingen, Tübingen, Germany; 21 Department of Surgical Oncology, University Medical Centre/Utrecht University, Utrecht, The Netherlands; 22 Department of Surgery, Rush University Medical Center Chicago, Chicago, IL USA; 23 Universitätsklinikum Frankfurt, Klinik für Allgemein-, Viszeral- und Transplantationschirurgie, Frankfurt, Germany; 24 Department of Surgery, Catholic University of Leuven, Leuven, Belgium; 25 Division of Surgery, Department of Hepatobiliary and Liver Transplant Surgery, St James’s University Hospital, Leeds, UK

## Abstract

**Background:**

Failure to rescue (FTR) is defined as the inability to prevent death after the development of a complication. FTR is a parameter in evaluating multidisciplinary postoperative complication management. The aim of this study was to evaluate FTR rates after major liver resection for perihilar cholangiocarcinoma (pCCA) and analyze factors associated with FTR.

**Patients and Method:**

Patients who underwent major liver resection for pCCA at 27 centers were included. FTR was defined as the presence of a Dindo grade III or higher complication followed by death within 90 days after surgery. Liver failure ISGLS grade B/C were scored. Multivariable logistic analysis was performed to identify predictors of FTR and reported using odds ratio and 95% confidence intervals.

**Results:**

In the 2186 included patients, major morbidity rate was 49%, 90-day mortality rate 13%, and FTR occurred in 24% of patients with a grade III or higher complication. Across centers, major complication rate varied from 19 to 87%, 90-day mortality rate from 5 to 33%, and FTR ranged from 11 to 50% across hospitals. Age [1.04 (1.02–1.05) years], ASA 3 or 4 [1.40 (1.01–1.95)], jaundice at presentation [1.79 (1.16–2.76)], right-sided resection [1.45 (1.06–1.98)], and annual hospital volume < 6 [1.44 (1.07–1.94)] were positively associated with FTR. When liver failure is included, the odds ratio for FTR is 9.58 (6.76–13.68).

**Conclusion:**

FTR occurred in 24% of patients after resection for pCCA. Liver failure was associated with a nine-fold increase of FTR and hospital volume below six was also associated with an increased risk of FTR.

Perihilar cholangiocarcinoma (pCCA) is a cancer arising of the extrahepatic bile duct, usually involving the biliary confluence. Often pCCA involves the portal vein (branches) or hepatic artery, and radical resection mostly necessitates a combination of resection of the bile ducts with (extended) hepatic resection, sometimes with vascular reconstruction.^1^ These procedures have a high rate of morbidity (overall complication rate 57% and major complication rate 40%) and a 90-day mortality of 10–18%.^2^

There is a growing demand for comparable and transparent health care outcomes that can help to compare the quality of care provided by different hospitals. In surgical oncology, numerous studies have attempted to clarify factors that may be important in comparing quality of hospitals, such as mortality, length of stay, costs per patient and hospital volume.^3,4^ Mortality is an often used performance indicator for surgical quality, but the incidence is low and dependent on multiple factors. These include patient factors such as age, tumor factors, preoperative management, surgical factors, postoperative complications, and their management. Failure to rescue (FTR) is defined as the death of a patient due to a major postoperative complication and has shown to be the main determinant for differences in mortality between hospitals rather than differences in complication rates.^5^ Parameters like FTR can be a quality parameter on how major complications are managed by the multidisciplinary team.^5–7^

Variations in mortality between hospitals after pCCA surgery may be explained by differences in patient selection, preoperative management (i.e., biliary drainage and portal vein embolization), the extent of surgery (i.e., the size of the liver remnant and vascular reconstructions performed), and surgical technique. Differences in FTR, rather than only the incidence of major complications, is an additional factor that can explain differences in postoperative mortality.^8^ Factors associated with the occurrence of FTR may include properties of the hospital and its organization; in particular patient volume and the presence of a multidisciplinary team for early detection and management of complications. This requires both expertise and teamwork of all specialties involved; nursing, surgical residents, gastroenterologists for endoscopic reinterventions, intensivists, and intervention radiologists for percutaneous reinterventions.^9^ FTR is especially relevant in pCCA surgery, due to the complex and abundant (major) complications including liver failure, cholangitis, bile leakage, and intra-abdominal infections, often inducing organ failure that requires multiple invasive interventions in a multidisciplinary approach.^10^

The objective of this study was to evaluate FTR rates after major liver resection for pCCA and analyze factors associated with FTR.

## Patients and Methods

### Patients

The need for ethical approval and individual informed consent was waived by the Institutional Medical Ethics Committee of the Amsterdam UMC. All patients undergoing a resection for pCCA were included in this study, except patients who had only undergone an extrahepatic bile duct resection, explorative laparotomy, or a liver transplantation. The definition of pCCA was a pathology proven or suspicion of a malignant biliary tumor originating at the hepatic ductal confluence between the segmental bile ducts and cystic duct. Patients who underwent resections for presumed pCCA were included from 27 participating centers collaborating in an international research group, the Perihilar Cholangiocarcinoma Collaboration Group. All centers included a consecutive series of patients operated between 2000 and 2023, but the inclusion period was not standardized across centers.

### Data Collection

Data were retrospectively collected by health care professionals per center independently. Length of follow-up was at least 90 days after surgery. Retrieved baseline characteristics were age, sex, ASA classification, jaundice prior to operation, biliary drainage, preoperative cholangitis, Bismuth classification, resection type, and vascular reconstruction.

### Outcome Parameters

All complications within 90 days after surgery were scored and classified according to the Dindo classification system, with grade III or higher considered as major complication. Perioperative mortality was defined as death within 90 days after surgery. The definition for FTR was death within 90 days after surgery in the presence of a major complication. Overall survival was defined as the time between surgery and death, or date of last follow-up. Preoperative cholangitis was defined as fever and leukocytosis requiring (additional) biliary drainage. Major liver resection was defined as resection of at least three Couinaud’s liver segments^11^. R0 resection margins were defined as tumor-free margins in all reported margins in the respective pathology reports. Hemorrhage, liver failure and biliary leakage were scored and classified according to the respective International Study Group of Liver Surgery (ISGLS) criteria, and only grades B and C were considered as clinically relevant.^12–14^

Hospitals were grouped into quartiles of the 90-day postoperative mortality rate. The rates of FTR and major complications were compared between these quartiles (groups).

### Predictive Factors for Failure to Rescue

Potential predictive factors for FTR were explored in logistical regression analysis were sex, age, ASA classification, BMI, jaundice at presentation, preoperative cholangitis, biliary drainage, Bismuth classification, portal vein embolization, bilirubin level at surgery, the type of resection, the year of surgery, and hospital volume, categorized in < 6 or ≥ 6 resections a year (based on annual median center volume in the cohort). A model with and without the addition of the variable postoperative liver failure was used.

### Statistical Analysis

Continuous data when normally distributed, were presented as a median with interquartile range as appropriate. Categorical data were presented as a frequency with percentage. A chi-squared test was used to compare categorical variable and Kruskall–Wallis or Mann–Whitney *U* tests for continuous variables.

Predictors of FTR were assessed in a standard multivariable logistic regression. Characteristics with a *P* value < 0.20 in a univariable analysis were entered into the multivariable model. Outcomes of the multivariable analysis were reported as odds ratio (OR) with the corresponding 95% confidence interval (CI). Two-sided *P* values < 0.05 were considered statistically significant. All statistical analysis were performed using SPSS (version 28.0 IBM, Chicago, IL).

## Results

### Patients and Outcomes

Out of 2492 patients who underwent surgery for suspected pCCA, 266 patients were excluded from further analyses; 70 did not undergo complete resection and 198 underwent only extrahepatic bile duct resection. The median number of patients included per center was 76 (44–95). Patients from centers with less than 30 inclusions were excluded (*n *= 38). The remaining 2186 patients were included in the analyses.

The median age was 65 (57–72) years and 1283 patients (59%) were male (Table 1). Preoperative biliary drainage was performed in 1802 patients (83%) and 466 (20%) experienced at least one episode of preoperative cholangitis. Preoperative portal vein embolization was performed in 787 patients (10%). The most frequently performed resection was extended right hepatectomy in 726 patients (33%), followed by left hepatectomy in 635 patients (29%), and right hepatectomy in 392 patients (18%).

**Table 1:** Baseline characteristics of patients who underwent major liver resection for Perihilar cholangiocarcinoma for those without complications, no failure to rescue and failure to rescue

	Full cohort *N *= 2186	No FTR *N* = 809	FTR *N *= 260	*P* value
Age, median (IQR)	65 (57–72)	64 (55–72)	68 (61–74)	< 0.001
Male sex, *n* (%)	1283 (59)	472 (58)	63.5 (64)	0.244
ASA 3/4, *n* (%)	833 (38)	285 (35)	112 (43)	0.003
Bismuth Type IV, *n* (%)	554 (25)	220 (27)	67 (26)	0.047
Jaundiced at presentation, *n* (%)	1740 (80)	618 (76)	217 (84)	0.002
Biliary drainage, *n* (%)	1802 (82)	696 (86)	231 (89)	< 0.001
Preoperative cholangitis, *n* (%)	466 (21)	198 (25)	74 (29)	< 0.001
Portal vein embolization,* n* (%)	430 (20)	193 (24)	73 (28)	< 0.001
Right liver resection, *n* (%)	1118 (51)	457 (57)	172 (66)	< 0.001
Extended liver resection, *n* (%)	1076 (49)	439 (54)	154 (59)	< 0.001
Caudate lobe resection, *n* (%)	1614 (74)	593 (73)	199 (77)	0.566
Portal vein reconstruction, *n* (%)	787 (36)	313 (39)	110 (42)	< 0.001
Hepatic artery reconstruction, *n* (%)	83 (4)	37 (5)	11 (4)	0.825
Liver failure, ISGLS grade B/C, *n* (%)	323 (15)	128 (16)	156 (60)	< 0.001
Bile leakage, ISGLS grade B/C, *n* (%)	432 (20)	325 (40)	66 (25)	< 0.001
Hemorrhage, ISGLB grade B/C, *n* (%)	212 (10)	91 (11)	61 (23)	< 0.001

### Complications and Mortality

A total of 1069 (49%) patients experienced a major complication and 275 (13%) patients died within 90 days after surgery. After a major complication, 260 patients died within 90 days, corresponding to an overall FTR rate of 24%.

**Fig. 1:**
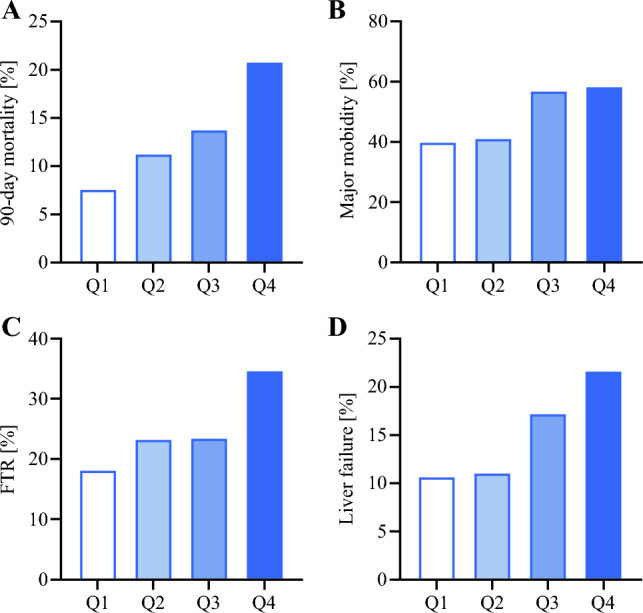
**A** The 90-day mortality rates in hospitals categorized in quartiles based on 90-day mortality rate. **B** Major complication, **C** FTR, and **D** liver failure rates based in the 90-day mortality quartiles

The major complication rate varied from 19 to 87% between hospitals and the 90-day mortality rate varied from 5 to 33%. The FTR ranged from 11 to 50% between hospitals. When centers were categorized into four quartiles based on 90-day mortality rate, the 90-day morality rates of the quartiles are 8%, 11%, 14%, and 21% (Fig. 1A). The major complication, FTR, and liver failure rates in these hospital quartiles are shown in Fig. 1B–D.

### Outcomes Based on Hospital Volume

The median annual hospital volume was 6 (4–7) patients. Hospitals that performed six or more resection per year were classified as high volume. The 90-day mortality rate was 12% in the high and 14% in the low volume hospitals (*P *= 0.059), while major morbidity rates were 51% and 46%, respectively (*P *= 0.040). This corresponded to an FTR rate of 23% in the high and 27% in the low volume hospitals (*P *= 0.078). Liver failure occurred in 17% of the patients operated by high and 11% in those in the low volume hospitals (*P* < 0.001).

### Factors Associated with FTR

At univariable analysis, age, ASA score of 3 or 4, jaundice at presentation, and right-sided liver resection were associated with FTR (Table 2). When included in the multivariable analysis, age, male sex, jaundiced at presentation, right-sided liver resection, and annual hospital volume ≤ 6 were individually associated with higher FTR (Table 2). When postoperative liver failure (grade B/C) was included in the multivariable analysis, jaundiced at presentation and right-sided liver resection lost their statistical significance, but liver failure was associated with an odds ratio for FTR of 9.58 (6.76–13.68).

**Table 2:** Uni- and multivariable analysis to identify factors associated with failure to rescue after major liver resection for perihilar cholangiocarcinoma

	Univariable		Multivariable model 1		Multivariable model 2	
	Odds ratio (95% CI)	*P* value	Odds ratio (95% CI)	*P* value	Odds ratio (95% CI)	*P* value
Age, (per year increase)	1.04 (1.02–1.05)	< 0.001	1.04 (1.02–1.05)	< 0.001	1.05 (1.03–1.07)	< 0.001
Male sex	1.24 (0.93–1.66)	0.144	1.24 (0.92–1.67)	0.159	1.42 (1.01–1.98)	< 0.043
ASA 3/4	1.62 (1.19–2.21)	0.002	1.40 (1.01–1.95)	0.043	1.26 (0.86–1.84)	0.234
BMI	1.01 (0.99–1.02)	0.588				
Jaundiced at presentation	1.69 (1.12–2.57)	0.013	1.79 (1.16–2.76)	0.009	1.47 (0.91–2.37)	0.118
Biliary drainage	1.29 (0.84–2.00)	0.246				
Preoperative cholangitis	1.29 (0.94–1.78)	0.112	1.23 (0.88–1.71)	0.225	1.12 (0.78–1.62)	0.544
Bismuth type IV versus I/II/III	0.94 (0.69–1.29)	0.708				
Portal vein embolization	1.22 (0.89–1.67)	0.227				
Bilirubin at surgery ≥ 50 µmol/L	1.25 (0.88–1.77)	0.219				
Right-sided liver resection	1.51 (1.12–2.02)	0.006	1.45 (1.06–1.98)	0.020	1.06 (1.74–1.50)	0.762
Extended liver resection	1.22 (0.92–1.63)	0.161	1.17 (0.85–1.62)	0.332	1.09 (0.76–1.55)	0.636
Caudate lobe resection	1.18 (0.83–1.69)	0.363				
Portal vein reconstruction	1.24 (0.93–1.66)	0.145	1.11 (0.81–1.52)	0.527	1.23 (0.87–1.76)	0.245
Hepatic artery reconstruction	0.91 (0.48–1.92)	0.906				
Surgery after the year 2010	1.01 (0.75–1.34)	0.968				
Annual hospital volume ≤ 6	0.76 (0.57–1.01)	0.060	1.44 (1.07–1.94)	0.017	1.95 (1.39–2.75)	< 0.001
Liver failure, ISGLS grade B/C	7.94 (5.81–10.84)	< 0.001			9.58 (6.76–13.68)	< 0.001

## Discussion

In this study of 2186 patients who underwent major liver resection for pCCA, major complication rate was 49%, 90-day mortality rate 13%, and FTR occurred in 24%. In a multivariate analysis for FTR, older patients, male patients, patients jaundiced at presentation, patients who underwent right-sided liver resection, and patients operated in a low volume center were at greater risk of FTR. Liver failure dominates the risk of FTR when included in the analysis.

FTR is increasingly used as a quality measure of surgery and is used to compare the outcomes of postoperative care across hospitals.^15,16^ It is hypothesized that early recognition and treatment of complications can reduce mortality.^17^ While complications are usually associated with mortality rates, FTR has been shown to account for a large part of mortality differences among hospitals.^18^ Prospective research supports this, such as the PORSCH trial in which an algorithm assisted in the early detection and treatment of pancreatic fistula after pancreatic resection and by that reduced 90-day mortality from 5 to 3%.^19^

The only other study to evaluate FTR in pCCA showed a FTR rate of 24%, comparable with the 24% in the current study.^8^ This study identified age above 65 years and right liver resection as predictive factors for FTR. These were also associated with FTR in this study along with several others, most likely due to the higher statistical power. When liver failure is included, jaundiced at presentation and right-sided liver resection no longer reach statistical significance in the multivariable analysis, most likely due to the higher probability of liver failure in the presence of these factors.^20^ The clear association of right-sided liver resection and liver failure with FTR is again a sign that preoperative estimation of postoperative liver function is essential, since there is no effective treatment for postoperative liver failure.^21^ It is essential to minimize the risk of (primary) liver failure by preoperative work up, and selection of patients for preoperative liver regenerative procedures or liver transplantation as alternative to resection. Remnant liver volume of at least 40%, a liberal approach to portal vein embolization, and quantitative liver function testing are all suggested to aid preoperative selection.^22–25^

Other larger studies including liver resection for all indications have identified hospital volume as an important predictor of FTR.^26–29^ All these papers report lower FTR rates in either higher volume centers or with higher surgeon volume. In the current study on resections for pCCA, annual hospital volume of six or more procedures was also associated with lower FTR. Liver surgery for pCCA is among the most high-risk procedures of any elective cancer surgery. The treatment of pCCA harbors challenges in accurate diagnosis and staging,^30^ biliary drainage and its associated complications,^31^ and the preoperative optimization of the future liver remnant volume and function.^32,33^ Therefore, it is likely that increasing experience in the treatment of pCCA will improve outcomes and the treatment should be concentrated in a limited number of centers. It seems reasonable to assume that adequate detection and timely treatment of adverse events is difficult when these procedures are performed less than once every two months.^34^ Experience with other hepatopancreatobiliary procedures will likely help to reduce FTR rates. ^35^

When based on the 90-day mortality quartiles, the hospitals in Q2 have similar major complications compared with Q1, but higher mortality, suggesting that these centers need to improve FTR by managing the complications. Aggressive treatment of infection and a low threshold to perform postoperative imaging studies might help to lower FTR, as has been shown in patients undergoing pancreatic surgery.^19^ The higher mortality in Q3 and Q4 seems related to the higher liver failure incidence, which suggests the improvements from these centers should come from preoperative selection of patient optimization. Besides the testing and optimization of the future remnant liver, avoiding biliary drainage in case of a large remnant and avoiding cholangitis in case of biliary drainage could help to reduce morbidity.^36^ Aggressive management infection could also help to avoid secondary liver failure, in which a vulnerable but sufficient liver is stressed by a complication and can lead to liver failure.^25^

This study has several limitations including the possible selection due to its retrospective nature. The timing of complications and data on the management of complications was not available. Therefore, it could not be shown whether the management strategy of complications influenced FTR. In addition, while 90-day mortality is an objective outcome, the scoring and grading of complications like liver failure is somewhat subjective, despite the ISGLS criteria. Therefore, the interobserver variability might have influenced the differences in liver failure rates between centers. In addition, to assess hospital volumes only, the number of resections for pCCA was used, not taking into account the volume and experience with other (major) liver surgery. Nevertheless, the used multicenter database is among the largest existing and this report is the first to investigate the hospital differences in FTR.

In conclusion, the FTR rate was 24% meaning almost one out of every four patients who experience a major complication after major liver resection for pCCA dies. Risk factors for FTR include higher age, males, ASA 3 or 4, jaundiced at presentation, right-sided resections, as well as an annual hospital volume below six procedures. Considering the rarity of the disease and complexity in the management of pCCA this is an argument to concentrate on the treatment of pCCA. The risk of FTR after major liver resection for pCCA is dominated by liver failure, which emphasizes the need for preoperative risk assessment and optimization of the function and size of the future liver remnant.
